# Supplementary material for: Survival Improvements in Advanced Hepatocellular Carcinoma with Sequential Therapy by Era
Source: Cancers (Basel). 2023 Nov 6;15(21):5298. doi: 10.3390/cancers15215298 (PMC10650854; doi:10.3390/cancers15215298)
Supplement: Supplementary file 1 [file cancers-15-05298-s001.zip › cancers-2701897-supplementary.pdf]

Table S1. Characteristics of BCLC-B patients

Variables		All	period 1 (2009-2013)	period 2 (2014-2018)	period 3 (2019-2022)	p-value
Number		125	26	55	44	
Age (years)	Median (range)	72 (48 to 89)	68 (48 to 82)	74 (53 to 82)	71 (50 to 89)	0.0505, 0.6090, 0.5077
Sex, *n* (%)	Female	20	3 (11.5)	6 (10.9)	11 (25.0)	0.184
	Male	105	23 (88.5)	49 (89.1)	33 (75.0)	
ECOG PS, *n* (%)	0	107	24 (92.3)	46 (83.6)	37 (84.1)	0.607
	1	18	2 (7.7)	9 (16.4)	7 (14.9)	
	2	0	0	0 (0)	0	
Body mass index (kg/m^2)	Median (range)	23.3 (11.2 to 33.0)	22.2 (15.7 to 32.7)	22.9 (17.3 to 32.7)	24.2 (11.2 to 29.6)	0.9954, 0.4990, 0.2329
Etiology, *n* (%)	Viral	90	23(88.5)	42 (76.4)	25(56.8)	0.012
	Non-Viral	35	3 (11.5)	13 (23.6)	19 (43.2)	
Child-Pugh class, *n* (%)	A	118	26 (100)	51 (92.7)	41 (93.2)	0.473
	B	7	0 (0)	4 (7.3)	3 (6.8)	
ALBI score	median (range)	-2.56 (-3.61 to -1.34)	-2.54 (-3.01 to -1.66)	-2.56 (-3.61 to -1.34)	-2.59 (-3.35 to -1.51)	0.7852, 0.5943, 0.9741
ALBI grade, *n* (%)	1 or 2a	91	22 (84.6)	37 (67.3)	32 (72.7)	0.282
	2b or 3	34	4 (15.4)	18 (32.7)	12 (27.2)	
Maximum intrahepatic tumor size, cm	median (range)	2.5 (1 to 25)	2.9 (1 to 13)	2.1 (1 to 6.6)	2.8 (1 to 25)	0.2557, 0.9910, 0.3074
Intrahepatic tumor number, *n* (%)	≤ 4	26	3 (11.5)	9 (16.4)	14 (31.8)	0.081
	≥ 5	99	23 (88.5)	46 (83.6)	30 (68.2)	

AFP (ng/mL)	median (range)	29 (0.9 to 5139)	29 (3.5 to 53139)	56 (1.9 to 39184)	15 (0.9 to 2627)	0.8709, 0.5406, 0.1675
AFP ≥ 400 ng/mL, *n* (%)	Absent	96 (76.8)	21 (80.7)	39 (70.9)	36 (81.8)	0.406
	Present	29 (23.2)	5 (19.2)	16 (29.1)	8 (18.2)	
The number of systemic therapy lines	1	90	25 (96.2)	39 (70.9)	26 (59.1)	0.0407
	2	20	1 (3.8)	10 (18.2)	9 (20.5)	
	3	7	0	4 (7.3)	3 (6.8)	
	≥ 4	8	0	2 (3.6)	6 (13.6)	

P-values for continuous variables are indicated between period 1 vs. period 2, period 1 vs. period 3, period 2 vs. period 3, respectively.

Abbreviations: ECOG PS, Eastern Cooperative Oncology Group Performance Status; ALBI, albumin-bilirubin; BCLC, Barcelona Clinic Liver Cancer stage; AFP, alpha-fetoprotein.

TABLE S2. Characteristics of BCLC-C patients

**Variables**		**All**	**period 1 (2009-2013)**	**period 2 (2014-2018)**	**period 3 (2019-2022)**	**p-value**
Number		211	60	77	74	
Age (years)	Median (range)	70 (19 to 89)	67 (36 to 84)	69 (19 to 89)	72 (46 to 87)	0.0950, 0.2562, 0.8685
Sex, *n* (%)	Female	34	6 (10.0)	16 (20.8)	12 (16.2)	0.241
	Male	177	54 (90.0)	61 (79.2)	62 (83.8)	
ECOG PS, n (%)	0	172 (81.5)	50 (83.3)	62 (80.5)	60 (81.1)	0.891
	1	31 (14.7)	8 (13.3)	13 (16.9)	10 (13.5)	

	2	8 (3.8)	2 (3.3)	2 (2.6)	4 (5.4)	
Body mass index (kg/m^2)	Median (range)	23.1 (15.8 to 31.8)	22.2 (16.4 to 30.2)	23.0 (15.8 to 30.9)	23.9 (17.6 to 31.8)	0.4090, 0.0179, 0.1957
Etiology, *n* (%)	Viral	147	49(81.7)	51 (66.2)	47(63.5)	0.047
	Non-Viral	64	11 (18.3)	26 (33.8)	27 (36.5)	
Child-Pugh class, *n* (%)	A	194	59 (98.3)	70 (90.9)	65 (87.8)	0.054
	B	17	01(1.7)	7 (9.1)	9 (12.2)	
ALBI score	median (range)	-2.53 (-3.58 to-0.91)	-2.39 (-3.28 to -1.24)	-2.55 (-3.58 to -1.25)	-2.59 (-3.35 to -1.96)	0.4240, 0.2008, 0.8489
ALBI grade, *n* (%)	1 or 2a	143	39 (65.)	52 (67.5)	52 (70.3.)	0.822
	2b or 3	68	21 (35.0)	25 (32.5)	22 (29.7)	
Maximum intrahepatic tumor size, cm	median (range)	3.0 (0 to 23)	2.6 (0 to 16.6)	2.8 (0 to 13)	4.1 (0 to 23)	0.9924, 0.2996, 0.0970
Intrahepatic tumor number, *n* (%)	≤ 4	130	33 (55.0)	45(58.4)	52 (70.3)	0.148
	≥ 5	81	27 (45.0)	32 (41.6)	22 (29.7)	
Macrovascular invasion, *n* (%)	Absent	129 (61.1)	35 (58.3)	50 (64.9)	44 (59.5)	0.685
	Present	82 (38.9	25 (41.7)	27 (35.1)	30 (40.5)	
Extrahepatic metastasis, *n* (%)	Absent	54 (25.6)	14 (23.3)	14 (18.2)	26 (35.1)	0.051
	Present	157 (74.4)	46 (76.7)	63 (81.8)	48 (64.9)	
AFP (ng/mL)	median (range)	102 (0.9 to 1057992)	108 (2 to 1057992)	32.5 (1.3 to 189050)	205.75 (0.9 to 129880)	0.8245, 0.9993, 0.7411
AFP ≥ 400 ng/mL, *n* (%)	Absent	128 (60.7)	36 (60)	52 (67.5)	40 (54.0)	0.239
	Present	83 (39.3)	24 (40)	25 (32.5)	34 (46.0)	
The number of systemic therapy lines	1	158 (74.9)	59 (98.3)	53 (68.8)	46 (62.1)	< 0.001

	2	30 (14.2)	0	13 (16.9)	17 (23.0)
	3	17 (8.1)	1 (1.7)	9 (11.7)	7 (9.5)
	≥ 4	6 (2.8)	0	2 (2.6)	4 (5.4)

P-values for continuous variables are indicated between period 1 vs. period 2, period 1 vs. period 3, period 2 vs. period 3, respectively.

Abbreviations: ECOG PS, Eastern Cooperative Oncology Group Performance Status; ALBI, albumin-bilirubin; BCLC, Barcelona Clinic Liver Cancer stage; AFP, alpha-fetoprotein.
